# Girl child marriage and the social context of displacement: a qualitative comparative exploration of Syrian refugees in Jordan and Rohingya refugees in Bangladesh

**DOI:** 10.1186/s12889-022-14832-z

**Published:** 2022-12-23

**Authors:** Jewel Gausman, Fauzia Akhter Huda, Areej Othman, Maysoon Al Atoom, Abeer Shaheen, Iqbal Hamad, Maysoon Dabobe, Hassan Rushekh Mahmood, Rifah Ibnat, Ana Langer

**Affiliations:** 1 Women & Health Initiative, Department of Global Health and Population, Harvard TH Chan School of Public Health, 665 Huntington Avenue, Boston, MA 02115 USA; 2 Maternal and Child Health Division, icddr, b, Dhaka, Bangladesh; 3 Department of Maternal and Child Health Nursing, University of Jordan, Amman, Jordan; 4 Center for Women’s Studies, University of Jordan, Amman, Jordan; 5 Community Health Nursing Department, University of Jordan, Amman, Jordan; 6 Jordanian Hashemite Fund for Human Development, Amman, Jordan

**Keywords:** Child marriage, Early marriage, Refugees, Jordan, Bangladesh, Syria, Humanitarian, Conflict, Social norms

## Abstract

**Background:**

There is growing global evidence that girl child marriage (CM) increases during humanitarian crises. Norms, attitudes, and policies that sustain CM are deeply entrenched within families and communities, and may be further exacerbated by conflict and displacement. The purpose of this study is to understand how the social and normative environment influences attitudes and practices related to CM in two diverse humanitarian settings.

**Methods:**

We held a total of eight focus group discussions, four in each country, with Syrian refugees in Jordan and Rohingya Refugees in Bangladesh. FGDs were conducted with fathers, mothers, and adolescent boys and girls.

**Results:**

Similar themes emerged from both settings. Participants discussed a desire to hold onto tradition in displacement and how norms are reinforced across generations. Social influence emerged in positive and negative ways, including peer pressure and conformity and the positive influence of host communities. In both settings, girls themselves described having little agency. Participants described resistance to change, which was exacerbated by conflict and displacement, though they discussed how social influence could be an effective way to challenge existing norms that drive the practice of girl child marriage.

**Conclusions:**

Our findings represent a more robust understanding of how norms operate within the social ecological system, and how they are reinforced across social relationships, offering an opportunity to more effectively challenge norms that sustain the practice of girl child marriage.

## Background

Child, early, and forced marriage (CEFM) is an understudied form of sexual and gender-based violence (SGBV) that has devastating consequences for women’s health throughout the lifespan. CEFM has been identified as an urgent public health challenge globally [1], and is linked to higher rates of early pregnancy and adverse maternal health outcomes [2], mental distress [3, 4], rape and physical abuse [5, 6], as well having a negative impact on child health [7], all of which are even more dire in humanitarian settings given the limited availability of health and social services.

Conflicts in both Syria and Myanmar have resulted in large-scale, humanitarian crises that have spilled into neighboring countries. In Myanmar, the Rohingya, a Muslim ethnic minority, have faced discrimination and violence for decades, including denial of citizenship and basic rights [8], with some arguing that the marginalization of the Rohingya began as far back as the British colonial era [9]. The current crisis began in 2017, when Myanmar’s army carried out a “clearance operation” in Rakhine State in which the Rohingya were subject to execution, rape, and destruction of their villages. As a result, nearly one million Rohingya refugees fled into Bangladesh. Currently, the vast majority of the Rohingya in Bangladesh (referred to as forcibly displaced Myanmar nationals, or FDMNs) live in dense camp settlements near Cox’s Bazar, the largest of which is the Kutupalong-Balukhali Expansion site, which hosts more than 600,000 refugees. Women and children make up the majority of refugees, putting girls at increased risk of child marriage, sexual exploitation, and other forms of sexual and gender-based violence. Structural barriers, including a shortage of schools, lack of investment, and host-country opposition, have resulted in limited educational opportunities for youth in the camps [10].

In Jordan, the humanitarian crisis began in 2011, at the start of the Syrian civil war, and includes more than 650,000 registered Syrian refugees. Some estimates put the total number of Syrian refugees in Jordan as high as 1.8 million, including those who are not formally registered as refugees. While Za’atri camp in Jordan’s Al-Mafraq governorate is one of the largest refugee camps in the world with a population of around 80,000 inhabitants, only 16% of the Syrian refugee population in Jordan resides in official camps. The majority live in urban areas, especially East Amman. Syrian refugees in both camps and community settings have disclosed high rates of sexual and gender-based violence. The Jordanian government and NGOs offer social services to the refugee population, including free access to state-run schools for registered Syrian refugee children, though significant barriers to access, including geographic, economic, and financial remain [11]. Unregistered refugees are extremely vulnerable and are often excluded from formalized governmental and institutional support.

Global evidence suggests that CEFM increases during humanitarian crises, especially during conflict-driven displacement [12]. The high prevalence of CEFM is an ongoing challenge among Syrian refugees in Jordan and FDMNs in Bangladesh, though the practice has existed historically in both populations. Recent estimates suggest that the prevalence of CEFM is now four times greater among the Syrian refugees in Jordan than was previously found in Syria [13]; while there is some debate as to the magnitude of the increase [14], studies have estimated the percent of girls married before the age of 18 years to be around 36.6% [15]. CEFM is also similarly thought to have increased among FDMNs in Bangladesh [16], though prevalence estimates remain sparse.

Religion, tradition, gender expectations, and the preservation of family honor have been found to be important drivers of CEFM among both Syrian refugees and FDMNs, while research shows that the influence of upstream factors that contribute to the ongoing practice of girl child marriage, such as economic vulnerabilities, safety concerns, and gender have been heightened by the challenges of displacement [12, 17–20]. Some recent research has also identified the influence that social pressure has on decisions relating to CEFM within humanitarian contexts, with some families fearing social ostracization and punishment if they do not conform to perceived behavioral norms [21]. Ensuring that humanitarian programs address the underlying drivers of CEFM that exacerbate harmful gender norms and foster resilience through transformative programming has been identified as a global priority; however, to date, there remains significant research and intervention gaps in this domain.

The purpose of this study is to explore how the social context in two diverse refugee populations has influenced the practice of girl child marriage from the perspectives of parents and youth. By investigating how factors within the social environment in these two diverse humanitarian settings gives rise to increased rates of CEFM, this study will contribute to generating practical evidence aimed at informing new and innovative interventions focused on transforming social norms that sustain gender inequality, and violence against girls and women.

## Methods

### Study design

This is a qualitative study in which we conducted a series of eight focus group discussions (FGDs), four in Bangladesh with FDMNs and four in Jordan with Syrian refugees. In each country, we held FGDs with the following population groups consisting of 5–7 members each: 1) fathers 2) mothers, 3) unmarried adolescent girls and young women and 4) unmarried adolescent boys and young men. Unmarried adolescent boys/girls and young men/women were eligible to participate if they were aged between 15 and 22 years. Fathers and mothers were eligible if they had adolescent children, married or unmarried, between the ages of 12 and 17 years. Participants were recruited by convenience at study sites.

We chose to use focus group discussions as our data collection methodology as they support group reflection and storytelling about collective experience and enable the researcher to understand shared and divergent perspectives and attitudes across a group. Further, they can improve discussion on sensitivity topics by enabling outspoken members to help make quieter group participants feel more comfortable speaking [22].

### Study setting

FGDs in Jordan took place at a community center in East Amman where community development activities regularly take place, including health, livelihoods, and civil society-related activities. The community center is frequented by a wide range of community member and is run by a long-standing, local, community-based organization. Amman is home to 195,303 registered Syrian refugees as of February 2021, though the actual population is likely higher as many are unregistered. East Amman is a densely populated, disadvantaged urban area heavily populated by Syrian refugees.

In Bangladesh, FGDs took place in Kutupalong-Balukhali Expansion site near Cox’s Bazar, which is a dense camp settlement hosting more than 600,000 FDMNs. Our study was conducted in a discrete camp within Kutupalong, with a total population of 26,434. Due to cultural norms, the FGD in Bangladesh conducted with young, unmarried girls was carried out in the home of a local community member, as young girls were not allowed to go out into the community. In both settings, all FGDs were conducted in a room of adequate size to ensure proper distancing, and all attendants were required to wear a mask, and used hand sanitizer prior to the FGDs to adhere to COVID-19 safety protocols. FGDs were facilitated by a trained and experienced facilitator of the same gender as participants.

### Ethical approval and consent

All individuals participating in the research activities provided verbal consent prior to participating. Parental permission was received for minors. Ethical approval was obtained from the Harvard TH Chan School of Public Health Institutional Review Board and necessary permissions were obtained locally to conduct study activities prior to their implementation. All methods in the study were performed in accordance with relevant institutional, national, and international guidelines. All FGDs were recorded. No personally-identifiable information was collected or retained with the transcripts. Audio files were destroyed after transcription to maintain participant confidentiality.

### Data collection and management

Participants were told that they were being invited to participate in a group discussion with the goal of understanding martial practices within their community. Data collectors were careful to use neutral language to describe the phenomenon of girl child marriage so as not to introduce outside bias into the discussion, given the cultural sensitivity of the practice, and cultural differences in the understanding, or lack thereof, of the phrase child marriage. Further, the study team held discussions after individual focus group discussions, and during data analysis, to engage in the practice of reflexivity. During self-reflection, researchers examined their interactions with study participants to identify potential interactions where their own individual position, assumptions or beliefs about child marriage may have influenced the research process.

Each FGD took between 1 and 1.5 hours. FGDs were conducted by individuals of the same sex as participants who regularly work on health and civil society programs targeting the study community, and were conducted in the language of preference of the study population: Arabic in Jordan and Rohingya in Bangladesh. All FGDs were audio recorded. The audio file was transcribed and translated from either Rohingya or Arabic to English.

### Data analysis

Each transcript was first read through multiple times then coded using a constant comparative approach to compare emergent to established themes, while simultaneously comparing findings across study populations and settings [23]. Analysis was conducted using Atlas.ti [24].

## Results

### Description of participants

In total, 51 people participated in the study. The breakdown of participants in each country by demographic group can be found in Table 1.

**Table 1:** Number of participants by country and demographic group

	Jordan (*n*)	Bangladesh (*n*)
Mothers	7	6
Fathers	7	6
Girls/Young women	6	6
Boys/Young men	7	6
Total	27	24

### Key themes

Our results centered on three overarching domains, and within each domain, several themes emerged. The first domain relates to the role of tradition, which includes the themes 1) Holding onto Traditions in Displacement and 2) Reinforcement Across Generations, and 3) A Deeply Embedded Practice. The second domain relates to the importance of social influence and expectations and includes the themes 1) Girls’ Muted Agency, 2) Peer Pressure and Conformity, and 3) The Role of the Extended Family and Community Members. The third domain focuses on opportunities for change, and includes the themes 1) Girls’ Education, 2) Social Diffusion, and 3) Positive Influence of Host Communities.

### Domain 1: the role of tradition

#### Theme 1.1: holding onto traditions in displacement

According to both Syrians and FDMNs, girl child marriage is a common practice that is deeply rooted in cultural tradition and centered on beliefs relating to purity, physical maturity, domestic responsibilities, and childbearing. Many FDMN participants in Bangladesh discussed the importance of a girl staying “pure” before marriage, and that a woman’s purity related directly to her age. Several participants described specific age cutoffs after which a girl is no longer considered to be fit for marriage, which was most often between the ages of 12 and 15 years. According to one FDMN father, “if a woman crosses the age of 15, then something is wrong with her physically or sexually. She is probably involved with another man or has diseases, due to which she is not getting married.” A FDMN boy said, “our parents marry our girls off early to save them from ‘nazar’ (bad glare).” Another young boy said going against the traditional practice has harsh consequences, “Because even if the girl herself, her parents and family members are not willing to marry her off before 18 years of age, the social norms make it very hard for her to get married afterwards into a good family.”

Syrian refugees focused on how others in their community would perceive a young woman who did not get married at what was considered to be an acceptable age. According to one Syrian boy, girl child marriage is commonly practiced “because of thoughts or ways of thinking, customs and traditions, and also the stigma. If the girl reaches 16 and she is not married, they will say she is a spinster”. A Syrian girl mentioned, “Some people think that if the girl doesn’t get married at the age of 14, she will get old and ugly and she will lose her beauty.” Syrian fathers also discussed concerns over girls becoming spinsters; however, they also emphasized how the practice of girl child marriage also satisfies social expectations related to domestic work and childbearing. One father explained, “He can also decide to take [a bride] younger than [18 years of age] because of pregnancy: when you marry a girl who is 20 or 25, there is short time to get pregnant while there is good time when you take a girl who is 15, 16, or 17.” Another father mentioned that a reason for girl child marriage in past generations was because, “the generation of our days got married between the ages of 14 and 15, because girls used to understand... know how to cook... know everything.” A Syrian adolescent girl had a similar reflection, “Because they think that [if] a girl can cook, clean, reach puberty and excel in housework, she’s capable of running a house.”

Both Syrians and FDMNs also described how the challenges of living in displacement has increased the reliance on traditional norms. One Syrian mother described “I think that we want to go back to how we’ve been raised, we used to live like that,” while a FDMN mother said that “Half of the people in the refugee camps understand the impacts of child marriage but the remaining half are still following the old rules.” A Syrian father described, “A war occurred and... [child marriage] is repeated again because of the situation we are forced to be in,” while another Syrian father said that war has also caused “a lack of young men in Syria. This encourages us to go back to child marriage,” due to the increased demand for child brides from men in the Gulf and the need and desire to marry off their daughters.

Participants from both populations also emphasized how economic and security-related challenges have contributed to the practice. A FDMN father described that the economic difficulties faced by many families in the camp is an important underlying issue, “Since they didn’t have sufficient space to have a proper standard of life, they would marry off their young girls in order to give their daughters and also themselves a better living condition.” One young FDMN boy described how girl child marriage is the direct result of both gender norms that limit women’s economic participation and the poverty faced by many in the camp setting saying that girls cannot participate in the “financial side of the family according to the social norms and rules of the Rohingya community...so [parents] marry them off early to reduce their responsibilities.” Syrian refugees in Jordan shared a similar story. One Syrian adolescent girl said that “Many people suffer from poverty, and decide to let the daughter get married so that her husband would be responsible for her.”

Ensuring girls’ safety also emerged as a key underlying issue in both settings. Several women in Bangladesh described situations where their daughters were kidnapped, and as a result they were then forced to marry them off. An adolescent FDMN boy said that, “parents believe that, if girls are married early, their safety is assured as she is no longer a single woman, and the potential risks like, being kidnaped, abused or harassed are reduced.” Similarly, a Syrian adolescent girl said safety is an important issue within their community, “Some people are afraid that something will happen to their daughter, so she should have support and someone to protect her.”

#### Theme 1.2: reinforcement across generations

Participants in both countries further described how the norms that perpetuate girl child marriage are passed from one generation to the next through normative expectations. An adolescent Syrian boy explained that “In the mentality of our Arab society, every behavior was followed from our ancestors from generation to generation,” while a Syrian adolescent girl said that the older generation tends to support the practice because, “What would make those who [previously] got married 15 or 20 years ago change their minds?” Another girl described how the younger generation learns to accept girl child marriage because they grow up expecting it, “it has been like this for a long time, we know that we get married in young ages, and our mothers and fathers got married at a young age.” A similar situation was described in Bangladesh. One FDMN adolescent boy said that, “The elders of the community belong in the old mindset, so they try to force the old norms over their children.”

A second route of intergenerational transmission was identified by participants specifically in Bangladesh. In Bangladesh, participants explained that the cycle of girl child marriage is self-perpetuating across generations due to an intergenerational cycle of poverty and lack of awareness. As a FDMN father said, “The children that come to the earth from early marriage...lack the care and education from their mother, as the mother herself is still a child, and unaware of the ways to take care of her own kids.” Similarly, an adolescent boy said that “I’m a child myself, how will I possibly take care of another child? How will I even know how to guide them [on] the right path and teach them, as I myself don’t know the right path.” Another adolescent boy focused on how the lack of educational opportunities available to girls further perpetuates the cycle of girl child marriage, saying “Give me an educated mother, I’ll give you an educated nation,” emphasizing that if a mother is educated, then they will make better decisions about their children’s future.

#### Theme 1.3: a deeply embedded practice

Participants in both settings described resistance to change as a barrier to reducing girl child marriage. A Syrian adolescent boy explained, “They might accept [that child marriage is a harmful practice], but the majority has a way of thinking that is related to customs and traditions and it is difficult to change,” while another Syrian boy comments, “the majority will not accept it, because they follow their customs and traditions, they feel safe with them, and it is difficult to change their way of thinking.” While participants in Jordan mentioned the need for legal loopholes to be closed as an important contributing factor to girl child marriage, they also emphasized that community practices and attitudes are as important as the laws in driving the continued practice of child marriage. As stated by a young Syrian girl, “You can’t change their minds, and no one abides to the law.” A Syrian mother reflected on how the community would be suspicious about outside intervention to change their customs and as a reaction, would likely show resistance to change, “Because there are people who think ‘who is this person, we are used to this, and no one will change us.’”

Several FDMN participants expressed a similar sentiment to those in Jordan about the difficulty involved in changing norms. One FDMN boy said that he tries to talk to the elders in the community, but no one listens to him because the acceptance of girl child marriage is so widespread. A FDMN father made a similar comment saying that while he tries to speak out about his views against child marriage, his community members do not pay attention to him and “they would rely on their social norms instead.” One FDMN father even suggested that the legal age of marriage should be reduced because of how strong the practice was within the community.

### Domain 2: the importance of social influence and expectations

#### Theme 2.1: girls’ muted agency

Adolescent girls in both settings described having limited ability to influence decisions relating to their marriage. Girls generally said that their fathers have the final say in matters related to marriage, regardless of what they want for their own life. As one FDMN mother explained, “Women forced to get married were told that they were given birth by their parents and so [their parents] are rightful decision makers of their marriage.” An FDMN adolescent girl expressed a degree of resignation in decisions relating to child marriage, “If the girls are pressurized by parents, they cannot resist ... other girls want to make the parents understand that early marriage is not good. Some parents listen, most don’t, and girls are bound to get married.” A similar dynamic was described amongst Syrian refugees. While some girls said that parents consult with their daughters about marriage, there was agreement that the girls had little influence in the ultimate decision. One young Syrian woman said, “It was my decision, but the very last word was up to my father.”

Another issue that limited girls’ agency in decisions relating to marriage is that several participants in both settings said that the girls making the decisions are too young to fully understand the consequences. As such, they initially agree with the decision to get married, but then realize that they have made a mistake. A Syrian mother said, “Sure, it was my decision, but I could not say no because I was too young. Then, when I saw that they agreed with me, I thought that I agreed [as well],” while an adolescent Syrian girl explained, “Well, a girl doesn’t know that she has to go to the court to get married, she is still underage, she does not know what marriage is and what responsibilities are. This means that she will not be mature enough, she will consider marriage as having fun. But this is wrong and indicates that she has no clue what marriage is.” In Bangladesh, a FDMN father expressed a similar sentiment, “[Girls] cannot assess the outcomes, because they are simply excited to get married.”

#### Theme 2.2: peer pressure and conformity

Both peer pressure and a desire to conform to social expectations were central concerns in both settings relating to decisions about when girls should get married. Syrians described feeling a desire to fit in to what they think everyone else is doing. One Syrian girl said that some girls do not want to be left out saying that some girls think that “Everyone is getting married at this age, why not me?” As Syrian father explained, “Of course, it becomes jealousy...” Unmarried girls also described being concerned about what married girls of the same age thought of them. A Syrian girl said that “Now those who got married before us think that we are spinsters.”

In both settings, there was also a focus on the negative consequences of not conforming to social expectations. A Syrian mother explained that some people are concerned that others in their community will think negatively of them if their daughters do not get married at the same age as other girls in their community, saying that “Some people depend on customs and traditions, if their daughter is 17 [years old] and the neighbor’s daughter who is aged 15 or 16 got married, they will have to marry their daughter too for her not to be a spinster”. A FDMN adolescent boy explained that “The badmouthing tendencies of the Rohingya society are a big problem because if a house has an unmarried girl who is over the age of 15, the society starts to create false rumors about the girl and her family, which make it very hard for them to maintain their social status. If a girl is “too old” then they have some problems for which their parents cannot get them married, and she’s being a burden over them.” A FDMN mother described a situation in which a boy and a girl wanted to get to know each other before marriage and wait until they turned 18 to get married, but “they were both constantly demeaned and humiliated, so they got married without finding another way,” while a FDMN father who chose to let his daughter wait she reached 18 years of age before getting married said that he has heard “some comments” from others in the community about his decision.

#### Theme 2.3: the role of extended family members and community members

In Jordan, all participant groups reflected on the role extended families and other community members play in decisions related to girl child marriage. While most indicated that the father or uncle has the final say in decisions relating to child marriage among girls, their decisions are heavily influenced by other extended family members. When asked if the final decision to marry a daughter actually rests with the girl’s parents, he said, “No, the decision is mostly on the grandfather and the older uncle. They’ve been consulted after all. We, as families, have hierarchies.” Similarly, a Syrian girl said that “I know someone who was opposed to early marriage, but then her older paternal aunt insisted and talked to her father. Two days ago, I heard she had a wedding. It was a shock because she was the one warning us about doing such thing.” A Syrian mother made a similar point and said that a man’s older brother and sister both have authority over him in decisions relating to his daughter’s marriage, leading another mother to exclaim, “Everyone interferes with the girl’s marriage!”

Syrian participants also discussed how other members of the community outside of the family are also influential in decisions relating to marriage. Several Syrian mothers said that neighbors and both the mother’s and father’s friends can all have a role to play, while another said that “The sheikhs may have an influence when they speak in mosques or consolation corners. In places like these, they may have an influence.”

### Domain 3: opportunity for change

#### Theme 3.1: girls’ education

Despite the difficulty in changing norms, some participants expressed some optimism that they are starting to see change. In both settings, participants attributed the changes they have observed to an increased understanding of the harmful consequences of child marriage among girls. In both settings, participants also discussed a strong relationship between norms related to girls’ education and child marriage; however, the nature of the relationship varied between the two settings. In Jordan, several participants discussed how they are seeing an increased emphasis on the importance of girls’ education, and more families are choosing to support their daughters in finishing their education prior to marriage. As one Syrian girl stated, “As for my family, we do not have to get married under the age of 18. Most of the girls in my family are studying. After we complete our studies, we will have the right age to get married.” Another girl commented, “Even in our family, I told you that my sisters got married under 18, but recently I received many proposals and my mother refused them telling me to complete my education and to get married afterwards. Ways of thinking can change.” However, the girls acknowledged that the acceptance of girls’ education is not uniform across society; “Certain social groups don’t support girls’ education, so once they get the chance to marry their daughter off, they will, saying that she will go to her husbands’ house eventually.”

In Bangladesh, norms related to girls’ education also appeared in direct opposition to the practice of child marriage among girls; however, participants described the grave lack of educational opportunities available in the camps as having a negative impact. Further, participants described how norms restrict girls’ movement outside the home, which makes it impossible for girls to attend school. All of the FDMN adolescent girls discussed a desire to attend school instead of getting married, but the girls said that they weren’t “brave enough” to tell their parents because of community norms that dictate that girls are not allowed to show their face in public. FDMN boys also focused on the lack of education as playing a central role in the wide acceptance of girl child marriage. One boy said that, “religion, society and the lack of something to do,” referring to the lack of employment and educational opportunities, are important factors that further perpetuate the desire among the youth in their community to get married early, especially among young men.

#### Theme 3.2: social diffusion

Despite the challenges associated with changing social norms, social influence was discussed by both Syrians and FDMNs as a possible way to change peoples’ minds. In Bangladesh, some participants referenced how norms could be questioned through certain influential social relationships. A FDMN father commented on the importance of social connections in changing people’s attitudes, saying that the poorest households are not connected to the most influential individuals who speak to the harms of child marriage among girls. Last, a FDMN adolescent boy mentioned that, “the best way to spread education is from beneficiary to beneficiary because peer learning is an effective way to spread knowledge.”

Several Syrian participants also discussed the importance of social influence in changing people’s attitudes and behaviors. A Syrian boy explained, “Our community is one-to-one, reachable through relationships and friends,” emphasizing the importance of social relationships in reinforcing or challenging people’s attitudes towards child marriage among girls. One Syrian boy described questioning others’ opinions related to child marriage saying, “we would have a little discussion, but at the same time, I must convince him in my way and convey the idea to him. Some of them support the idea, and sometimes we use religious proverbs.” Adolescent girls described how parents may be convinced to change their minds when challenged by influential family members who do not support child marriage among girls, saying, “Maybe at this point the parents will reconsider the matter.” Another adolescent girl described how people are greatly influenced by what they think that the majority of other community members are doing, “I’m positive that people will act like the others in the governorate and will get married after the age of 18, and if one governorate is convinced, then the country will [also be convinced], then customs will change among people.”

In both settings, young men were specifically mentioned as being particularly influential in discussions about CEFM. An FDMN boy said that, “the children are...learning to teach [the elders] the right information about these issues,” and that he is willing to go against his parents to keep his sister unmarried until she decides that she is ready. In Jordan, a Syrian father also referenced the importance of young men in changing attitudes saying, “Who can prevent the idea of child marriage in the Syrian society is young men.”

Young men themselves expressed being influenced by seeing the harmful effects of child marriage on women’s health, but also in terms of limiting women’s potential. Several FDMN boys mentioned that seeing the negative effects of child marriage among girls first-hand has caused them to speak out against it. Similarly, in Jordan, a Syrian boy said that “From my point of view, it is a big problem, because most of the cases of child marriage cause problems that lead to injuries and death, during childbirth, for example. If one of my friends told me that he wants to marry a girl who is 16 or 17 years old, I would tell him that she is young and he will destroy her, and I would try to give him the correct opinion.” Another Syrian boy said, “I am not the kind of person who would be forced to take a child under the age of 16. In my view, she’s a child, I would deprive her of her education and prevent her from her future. Her future may be better than mine. She can have a higher degree. I could be a doctor, and she might be better than a doctor.”

#### Theme 3.3: the positive influence of host communities

Host communities were also described as being influential in reducing people’s acceptance of child marriage among girls. Several participants noted that they believe that the members of their community place great importance on the host population’s opinions. One FDMN adolescent girl described how, “parents would listen to host communities if awareness of child marriage is taught to them, but [they] won’t listen to their daughters.” While another girl said that host communities are in a position to challenge the norms that restrict girl’s movement and limit their access to education by stating, “host communities can make parents understand that girls should also be allowed to go out and educate themselves.”

In Jordan, participants also commented on the role of host communities in changing attitudes and practices related to girl child marriage. A Syrian mother said, “as Syrians, we used to marry the girls off at a young age, and by the time we came to Jordan we noticed that a large number of Syrians are refusing early marriages...” When asked if this started when she came to Jordan, she added, “Yes, I frankly felt that this culture and awareness spread among people, and I felt that people did not marry their daughters off until they had completed their education and established terms to be committed upon. These views are spreading among Syrians and the impact is positive.” Similarly, a Syrian girl said that “When people were in Syria, everyone got married at the age of 18 or less, but when we came here, our way of thinking changed. It is not like the old traditions and customs, forcing people to get married.”

## Discussion

This study points to how the social context in refugee settings may contribute to the ongoing practice of girl child marriage, putting girls, young women, and their children at increased risk of a wide range of poor health outcomes that are further exacerbated by the difficult living situation that many refugees face in displacement. Some of our results reinforce those from previous research, primarily among Syrian refugees; however, our study also provides new insight into how leveraging the social ecology may provide programs with an opportunity to bring about change. Further, while our results point to more similarities than differences in terms of how the social context drives child marriage within the two diverse refugee populations examined in the study, we also identify important differences. Both add to the literature by providing a unique cross-comparative perspective between humanitarian settings.

The unique challenges associated with humanitarian crises and displacement add to the complexity of the role of norms in influencing behavior, while potentially increasing their salience as a target for intervention. Norms, attitudes, and practices that support CEFM are deeply entrenched within families and communities in populations where CEFM is practiced [25–27]. Our results build on those from previous research that suggests the new social environment brought about by displacement may cause these existing norms to evolve in both positive and negative ways [28], causing them to interact with the new contextual environment in ways that are different from the pre-crisis reality. As with past research conducted with Syrian refugees in Egypt, we find that in both of our study populations, gender inequitable norms that aim to control young women’s behavior appear to be brought to more extreme manifestations as a result of displacement due to weak legal frameworks, increased economic fragility, lack of educational opportunities, and urgent concerns over girls’ physical safety [28].

A common underlying theme across both study population was that social norms were seen by many of the participants as a key barrier that has limited the success of previous efforts to reduce girl child marriage. In both settings, participants also generally described having little success in their individual efforts aimed at convincing other members of their community about the negative consequences of CEFM on their own; however, they emphasized what they believed to be the important role of social diffusion in changing norms. Previous research conducted among Syrian refugees in Lebanon also suggests that girls and women may benefit from interventions focused on changing attitudes related to gender equity, but that such interventions would likely have limited impact unless coupled with interventions to target widespread normative change within families and communities [29].

Normative research seeks to understand what behaviors are thought to be typical (descriptive norms), what behaviors are expected (injunctive norms), and how social sanctions disincentivize people from deviating from the norm. While the specific norms that lead to child marriage across both study populations in our research vary, our results points to more similarities than differences related to the way in which norms operate with the social context to influence child marriage within these two diverse settings. In both settings, participants emphasized how descriptive norms and injunctive norms operate simultaneously to uphold the continued practice of child marriage. Descriptive norms driving the continued practice of girl child marriage appeared in both study sites with participants describing how common the practice is within the community. At the same time, the role of tradition in driving the practice of child marriage created a social expectation, or injunctive norm, among both Syrians and FDMs. Both populations described being concerned about social sanctions or negative consequences should girls wait too long to get married. Such concerns were expressed by family members across both sites and echoed by the girls and young women who are the targets of child marriage as well.

Identifying and exploiting areas of discordance and misalignment between the types of norms and individual attitudes represent areas for intervention [30]. While many programs aim to address social norms in humanitarian settings, few norms-based interventions to date have been identified to reduce CEFM [31]. Rather than pointing to the limited effectiveness that norms-based interventions have shown to date, however, we believe our results point towards the need for more robust research on the specific norms that govern behavior and the social and structural mechanisms through which norms are transmitted to identify more salient approaches to engender normative change.

The results of this study also speak to the need for more research on understanding which relationships between individuals in the community are the most influential and which can be leveraged to challenge community norms. Participants described that a wide variety of social relationships are instrumental in influencing decisions about marriage, ranging from immediate and more distal family members, peers, neighbors, and religious leaders. Existing research on CEFM often has often focused on the role of immediate family members in decision-making aligned with traditional familial hierarchies; however, a deeper understanding as to how a wide variety of social contacts influence decisions relating to CEFM, including women and adolescent girls’ own peer networks, is needed [21]. Prior research has suggested that Syrian refugees have been influenced by Jordanian women in their communities with regard to other social and economic issues [32]. Building on this, our results illustrate that such influential relationships may also be important to leverage in the case of child marriage.

Questions have emerged in the literature about how norms interact with girls’ own agency with regard to child marriage in different contexts. Traditional framing of girls’ agency assumes a positive, linear relationship between knowledge, agency, and girls’ ability to resist CEFM [33]. Research on girls child marriage in Latin America suggests that expressions of agency among girls and young women may result in them being willing participants in marriage rather than resisting the practice [34]. Girls and young women in our study described being convinced by their families and peers that early marriage is a desirable outcome due to injunctive norms that ascribe rewards or status to girls upon marriage before being able to fully assess the consequences. Such norms result in manifestations of agency among girls that ultimate accommodates the practice.

Our results raise important new questions of how more complex mechanisms related to informal behavioral control and social support operate to influence acceptance of and decisions related to CEFM. Participants described the negative responses that other members in their community have to girls’ who delay marriage that could have negative repercussions on a girl’s future marriage prospects, a family’s relationship with others in the community, and thus their ability to rely on other community members as resources for both social and material support. They also discussed at length how individual’s attitudes and behaviors related to girl child marriage are shaped by the different people around them. Qualitative research in Jordan has emphasized that shifting social networks during displacement have likely had an impact on decisions relating to CEFM, due to real and perceived vulnerabilities arising from a lack of social support, as most social support centered on the extended family network in Syria whereas in Jordan, neighbors are often unknown [35]. Syrian refugees in other studies have also described that help-seeking behavior and coping strategies primarily rely on informal support networks within their families and their communities, while little support from outside, institutional resources was described as meaningful [36].

As such, normative influence may have an even more pronounced impact on decision-making as displaced individuals seek to fit in to new networks and adapt to new normative structures. Other research with Syrian refugees in both Jordan and Lebanon found that exposure to more liberal norms within host communities caused families to turn to child marriage as a way to protect their daughters [37, 38]. While these findings seem to contrast with the positive influence that we found host communities to have on shifting norms and attitudes away from girl child marriage, it may be instead that such divergent findings are really describing different manifestations of the same underlying phenomenon. Other research has identified an association between difficult living circumstances, feeling hopeless about the future, and a lack of integration with host communities among Syrian refugees in Jordan, and that this was especially pronounced in camp-based populations [33]. In our study, participants in both Jordan and Bangladesh described the positive influence of host communities as occurring through social connections and active discussion, rather than through passive observation. It may be that the felt need described by refugees to protect their daughters from a more liberal host community was the result of observing such behaviors from a distance among refugees with little social connection to the host community. Contextual differences between the study populations also reflect what was observed in other studies; camp-based populations may have less opportunity to engage with the local host community, thus intensifying resentment and hopelessness. While subtle, in our study, participant reflection about the role of host communities in changing attitudes appeared to be more concrete and based on past experience among Syrians (largely community-based) as opposed to more theoretical among the entirely camp-based population of FDMNs. More research should be conducted to better understand how isolation from, versus deeper social integration with, the host community may affect norms related to child marriage within displaced populations, and whether diversifying refugee social networks in these settings could be a viable pathway for intervention.

Important contextual differences also exist between the two study populations that may also give rise to differences in how the social context influences norms related to girl child marriage. In both settings, girls face extreme movement restrictions due to cultural norms and fears over security; however, Syrian girls in Jordan have access to the host country’s education system versus FDMN girls having no access to education in Bangladesh. For FDMNs, it appears that this combination further reinforces and validates the norm of controlling girls’ movement. Conversely, in Jordan, our results suggest that some families were beginning to prioritize education over marriage for their daughters. Other research has argued that access to girls’ education may nurture the development of collective capabilities among adolescent girls to overcome the structural ‘unfreedoms’ that force them into marriage [39]. Taking this perspective into consideration with our study’s results, it may be that enabling girls to access the host country’s education system helps them to develop these capabilities, while simultaneously providing an opening in society to do so by fostering the development of more integrated social networks between refugee and host communities. Understanding the role of social networks in CEFM may be an important new area of research in both settings under study, as well as in other refugee populations.

This study is subject to several strengths and limitations. First, even though our study sample is diverse, the number of participants in each demographic group is relatively small. Further, the geographic scope of our study is limited and focuses on specific communities. Broad generalizability is not typically a goal of qualitative research. Therefore, we believe that the deep look into local perspectives that our study offers resulting from its focus on specific populations and geographies is a strength rather than a limitation. Further, looking across our FGDs, our results showed evidence of saturation, with no new themes emerging from across FGDs, implying that our study population was sufficiently large. Saturation is thought to be an important element related to the underlying validity of qualitative research; while observing similar themes emerge using different data sources (in our case, distinct research settings) may imply reliability in the results [40, 41]. Second, while there was robust discussion within each FGD, CEFM is a sensitive topic in both study communities and thus social desirability bias may have played a role in participants’ responses. To reduce this possibility, FGDs were conducted by local individuals to improve the validity of the data by making participants less suspicious of an outside agenda in driving the discussion. At the same time, it is important to note that members of the study team generally held beliefs that child marriage is a practice that should be eliminated. While researchers were careful not to let this perspective emerge during the data collection and analytical process, and the team critically assessed the impact that such a position may have on the research, it is possible that despite our efforts, this may have increased the potential for social desirability bias to emerge in participant responses and thereby influence our results. Finally, we explore child marriage as it occurs primarily among girls and through the perspectives of children, young adults, and parents. Child marriage among boys is an important problem as well, though it occurs to a lesser magnitude in the populations examined here, and should be the subject of additional research. While our study includes a variety of individual perspectives to explore the social environment, we were unable to include other perspectives from within the community from the institutional perspective, such religious, education, and health-focused institutions, though we consider this to be outside the scope of our research.

## Conclusions

CEFM is a complex public health and human rights issue that is exacerbated in humanitarian settings. Despite the differences between the FDMN and Syrian refugee populations, important similarities emerged from our results across sites that may be valuable for research and programs within Bangladesh and Jordan, but also in other humanitarian settings with displaced populations globally where CEFM is practiced. While existing research has focused on identifying many of the important social and structural drivers of CEFM, robust research to explore the mechanistic pathways through which social norms influence attitudes and behaviors related to CEFM is needed to inform interventions and related health outcomes, especially in humanitarian settings.

## Data Availability

The datasets analyzed during the current study are not publicly available due to human subject’s protections, but are available from the corresponding author on reasonable request.

## Abbreviations

CEFM: Child, early and forced marriage
FDMN: Forcibly displaced Myanmar Nationals
FGDs: Focus group discussions
